# *Leptospira* diversity in animals and humans in Tahiti, French Polynesia

**DOI:** 10.1371/journal.pntd.0005676

**Published:** 2017-06-28

**Authors:** Vanina Guernier, Vaea Richard, Tuxuan Nhan, Eline Rouault, Anita Tessier, Didier Musso

**Affiliations:** 1 Institut Louis Malardé, Papeete, Tahiti, French Polynesia; 2 Australian Institute for Tropical Health and Medicine (AITHM), JCU, Townsville, Australia; Yale University Yale School of Public Health, UNITED STATES

## Abstract

**Background:**

Leptospirosis is a highly endemic bacterial zoonosis in French Polynesia (FP). Nevertheless, data on the epidemiology of leptospirosis in FP are scarce. We conducted molecular studies on *Leptospira* isolated from humans and the potential main animal reservoirs in order to identify the most likely sources for human infection.

**Methodology/Principal findings:**

Wild rats (n = 113), farm pigs (n = 181) and domestic dogs (n = 4) were screened for *Leptospira* infection in Tahiti, the most populated island in FP. Positive samples were genotyped and compared to *Leptospira* isolated from human cases throughout FP (n = 51), using *secY*, 16S and *LipL32* sequencing, and MLST analysis. *Leptospira* DNA was detected in 20.4% of rats and 26.5% of pigs. We identified two *Leptospira* species and three sequence types (STs) in animals and humans: *Leptospira interrogans* ST140 in pigs only and *L*. *interrogans* ST17 and *Leptospira borgpetersenii* ST149 in humans and rats. Overall, *L*. *interrogans* was the dominant species and grouped into four clades: one clade including a human case only, two clades including human cases and dogs, and one clade including human cases and rats. All except one pig sample showed a unique *L*. *interrogans* (*secY*) genotype distinct from those isolated from humans, rats and dogs. Moreover, *LipL32* sequencing allowed the detection of an additional *Leptospira* genotype in pigs, clearly distinct from the previous ones.

**Conclusions/Significance:**

Our data confirm rats as a major potential source for human leptospirosis in FP. By contrast to what was expected, farm pigs did not seem to be a major reservoir for the *Leptospira* genotypes identified in human patients. Thus, further investigations will be required to determine their significance in leptospirosis transmission in FP.

## Author summary

Leptospirosis, a zoonosis caused by *Leptospira* spp. bacteria, is an important but neglected disease in the Pacific region. The bacteria can lead to human infections, either through direct contact with infected animals or through water and soil contaminated with urine. Although animals play a key role in the transmission of the disease, the relative importance of animal reservoirs for human leptospirosis infections in the Pacific have not been investigated. In French Polynesia, the animal species acting as sources for human infection are unknown. In our study, we isolated leptospires from rats, farm pigs and pet dogs from Tahiti, the most populated island of French Polynesia, and compared them to those isolated from human leptospirosis cases. The types of leptospires identified in human leptospirosis cases were also detected in rats and dogs, whereas leptospires found in pigs were genetically different. These results indicate that rats are a major potential source for human leptospirosis in Tahiti. Further investigations will be required to clarify the relative contribution of dogs and pigs to human leptospirosis in French Polynesia, to properly design or adapt interventions to reduce the burden of human leptospirosis.

## Introduction

Leptospirosis is a disease caused by leptospires, bacteria belonging to the order Spirochaetales, family *Leptospiraceae*, genus *Leptospira* [1]. Leptospirosis is the most widespread zoonosis worldwide [2], but a neglected disease in most of the tropics, especially in the Pacific region [3]. Incidence of leptospirosis range from 0.1 to 1 cases per 100,000 inhabitants per year in temperate climates, 10 to 100 cases per 100,000 in the humid tropics, to over 100 cases per 100,000 in high risk groups and during outbreaks [4,5]. The clinical spectrum of human infections ranges from mild flu-like illness to severe or even fatal outcome. The genus *Leptospira* is divided into 22 species classified into saprophytic, intermediate and pathogenic groups [6]. Numerous animals, including rodents (considered as the main reservoir), domestic mammals (including livestock) and wildlife, are reservoirs for leptospires [7]. Leptospires are maintained in the proximal tubules of the kidneys of infected animals (chronically in animal reservoirs, or temporarily during acute infection) and excreted in urine, from which they contaminate soil, surface water, streams and rivers. Humans are infected through direct contact with urine or tissues from infected animals, or indirectly via a contaminated environment, particularly if there are abrasion or cuts in the skin. Prolonged immersion in, or swallowing of, contaminated water can also increase the risk of infection [2].

French Polynesia (FP) is part of the 22 Pacific Countries and Territories, with a population of ~270,000 inhabitants living on five archipelagos (Society, Marquesas, Tuamotu, Gambier and Austral Islands) and about 70% residing on the island of Tahiti. The annual incidence of leptospirosis in FP range from 30 to 55 per 100,000, with one to four fatal cases per year [8,9]. The incidence of leptospirosis in the Pacific region is not well-documented, mainly due to a lack of diagnostic facilities [10,11]. In addition, leptospirosis is sometimes misdiagnosed as other infections, especially arbovirus infections [12]. Data on the *Leptospira* serovars circulating in humans in FP are scarce. The most recent serotyping information was from cases confirmed in 2007–2010 from FP; 100 isolates were identified as serovars Icterohaemorrhagiae (serogroup Icterohaemorrhagiae) (50%), Australis (serogroup Australis) (32%), Canicola (serogroup Canicola) (7%), Ballum (serogroup Ballum) (4%), Hebdomadis (serogroup Hebdomadis) (4%) and Weilii (serovar Topaz serogroup Tarassovi) (3%) [8]. However, we cannot rule out that other serovars may be present as serotyping was available from only 20% of cases, and because only the serovars included in the panel of antigens can be detected. In addition, molecular studies have never been conducted in FP, so the genetic diversity of *Leptospira* is unknown.

Specific serovars are more commonly associated with particular reservoir hosts, such as Ballum with mice, Canicola with dogs, or Icterohaemorrhagiae with rats [13]. For this reason, rats, which are very common in FP, were believed to be the main reservoir of leptospires in this country. However, results of epidemiological surveys have suggested that other species might also act as important reservoirs, e.g. dogs and pigs in Society Islands, wild pigs in the upper islands, horses and goats in Marquesas [14]. Until now, animal leptospirosis studies had never been conducted in FP, and the role of animal reservoirs has therefore remained speculative.

The identification of animal carriers and reservoirs of pathogenic leptospires in FP is key to understanding the most probable routes of human exposure, and to recommend public health interventions to reduce leptospirosis disease burden. The aim of our study was (i) to estimate the prevalence of *Leptospira* carriage in rodents, pet dogs and domestic pigs, and (ii) to identify the *Leptospira* genotypes circulating in humans and animals, and thereby determine which host species may serve as important reservoirs for pathogenic leptospires in FP.

## Materials and methods

### Sample collection of human sera

Human sera (n = 44) were retrospectively analysed from leptospirosis cases confirmed by molecular diagnosis from January 2014 to April 2015 at the medical diagnosis laboratory of the “Institut Louis Malardé” (ILM). Cases originated throughout FP: Tahiti (n = 15), Raiatea (n = 9), Moorea (n = 8), Tahaa (n = 7), Huahine (n = 3). The island of origin was missing for two samples. Human sera from seven confirmed cases from November 2011 to January 2012 were further added to our analysis to match the collection date of pig samples. Those cases originated from Tahiti (n = 3), Raiatea (n = 1) or Moorea (n = 3).

### Sample collection of animal specimens

**Fig 1:**
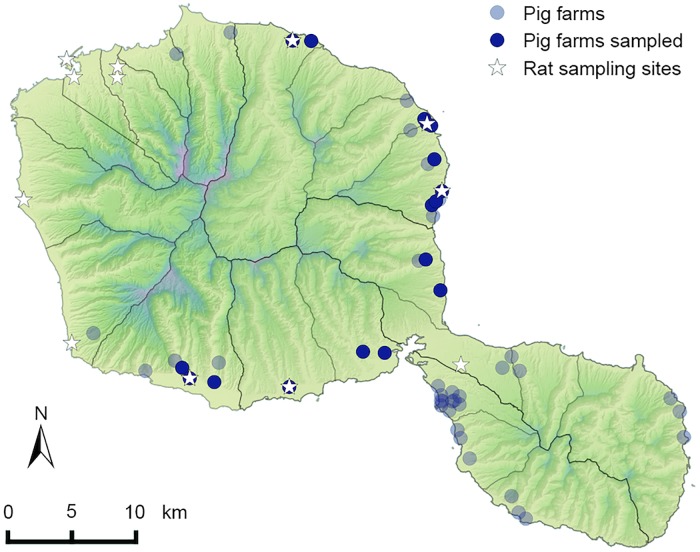
Geographic localization of piggeries and rat sampling sites on Tahiti Island. The pig kidneys tested for *Leptospira* infection originated from 17 herds representing 16 farms. Rats were trapped at 12 sampling sites, including five farms from which pig kidneys originated.

Clinically healthy pigs (*Sus scrofa*; n = 181) originating from 17 herds representing 16 farms across Tahiti (Fig 1) and one farm in Moorea (Society Islands) were included. Their kidneys were collected from November 2011 to March 2012 at the slaughterhouse of FP (SAEM Abattage de Tahiti) in collaboration with the Agriculture Department of FP.

Rats (n = 113) were trapped from 12 sample sites throughout the island of Tahiti (Fig 1) from February to April 2015. Five of the sampling sites were farms from which previously collected pig kidneys originated. Trapping was conducted with wire cage live traps baited using either grilled coconut pieces or a mixture of peanut butter and canned sardine oil. At each sampling site, 40 traps were placed in the afternoon, in line when possible and at least 15 meters apart; trapped animals were collected the following morning and brought back to the laboratory for dissection. If less than ten individuals were trapped at one site, traps were left open in the same place for a second night.

Dog sera (n = 2) were collected from animals with clinical suspected leptospirosis. Kidneys from other dogs (n = 2) were collected by a veterinarian immediately after the animals died from leptospirosis.

### Rat identification

Rat species were identified using morphological criteria [15] followed by a molecular confirmation by amplification of the COI gene with the primers BatL5310 / R6036R [16].

### Ethics statement

The study was approved by the “Ethics Committee of French Polynesia” (n°61/CEPF). All animal procedures carried out in our study were performed in accordance with the European Union legislation for the protection of animals used for scientific purposes (Directive 2010/63/EU). Human sera were clinical samples collected for diagnostic purposes, and all patient data were anonymized prior to analysis.

### Total nucleic acids extraction

All kidney samples were dissected aseptically spanning the corticomedullary junction to collect 20–25 mg of tissue. Tissue sample was lysed for 30 minutes at 56°C with 50 μl of proteinase K (600 mAU/ml, Novagen) and 50 μl of TRIS-HCL solution at 20mM, pH 8.3, 0.5% sodium dodecyl sulphate. Samples were vortexed every 10 min and then centrifuged at 9,000 rpm for 3 min to collect the supernatants.

Total nucleic acids (i.e. both DNA and RNA) were extracted from 100 μl of kidney supernatants and from 200 μl of human and dog sera using the NucliSENS easyMAG automated platform following the manufacturer’s instructions (BioMérieux, France). The elution volume per sample was 50μl.

### Detection of *Leptospira* spp.

Previous studies have shown a better performance of RT-qPCR compared to qPCR for *Leptospira* detection [17–19]. In addition, RT-qPCR allows the detection of both DNA and RNA, increasing the overall sensitivity of the assay [20]. Thus, we performed qualitative RT-qPCR to detect (i) the 87-bp region of *rrs* (16S) gene with the primers Lepto-F, Lepto-R, Lepto-S probe for the rat samples [21], or (ii) the 241-bp region of *LipL32* gene with the primers LipL32-45F, LipL32-286R and probe LipL32-189P [22] for the other samples. Preliminary sensitivity assays were conducted to select the gene to be targeted for *Leptospira* detection in the samples (S1 Table). Amplifications were done using the iScript One-Step RT-PCR kit for probes or the IQ Supermix (Bio-Rad Laboratories, France). PCRs were carried out on CFX96 Touch Real-Time PCR Detection System instrument (Bio-Rad Laboratories, France) using the following conditions: initial denaturation at 95°C for 3 min, followed by 45 cycles of denaturation for 3 sec at 95°C and annealing/elongation for 15 sec at 58°C. PCR detection in animal samples was performed in duplicate, or in triplicate when necessary. Using a conservative approach, samples were considered positive when the cycle threshold (*Ct*) value was inferior to 41 cycles for two replicates, keeping in mind that detection rates may be underestimated. All PCRs were run with negative and positive controls (*L*. *interrogans* serovar Australis and *L*. *kirschneri* serovar Grippotyphosa reference strains).

### *Leptospira* genotyping

**Fig 2:**
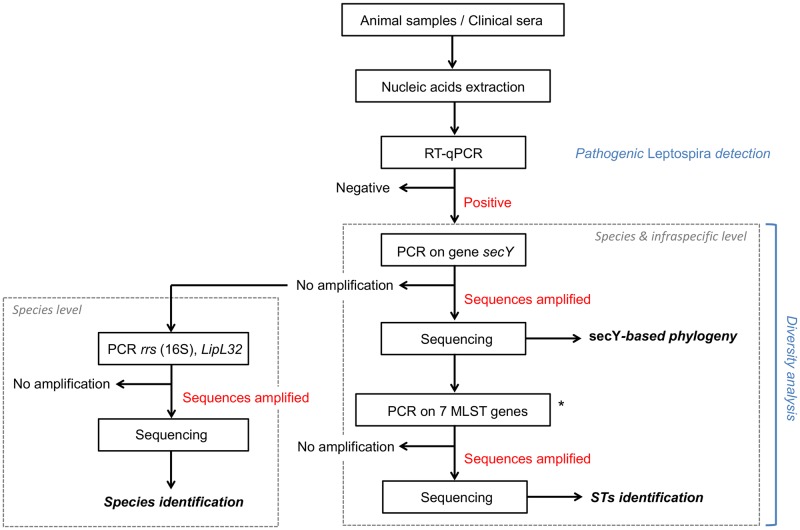
Flowchart describing the steps of the molecular analysis. Methods used to detect and to identify *Leptospira* species and genotypes from samples collected in French Polynesia are detailed. Seven-loci MLST was attempted only for a selected subgroup of the animal and human samples successfully amplified on *secY* locus using MLST scheme #1.

*Leptospira* positive samples were subjected to different genotyping methods in order to determine and to compare the *Leptospira* genetic diversity at the specific and infra-specific levels (see Fig 2). First, they were tested by *secY* sequencing, a gene which has been shown to be suitable for species identification and phylogenetic studies [23,24]. Primers secY-F / secY-R were used to amplify 549-bp fragments [25]. When *secY* PCR amplification failed, sequencing of alternative short regions was performed to allow *Leptospira* species identification: a 331-bp sequence from the *rrs* (16S) gene using primers LA / LB [26] and if necessary a 241-bp sequence from the *LipL32* gene using primers LipL32-45F / LipL32-286R [22]. All PCRs were run with negative and positive controls as detailed above.

Based on the observed diversity within the *secY* gene, multilocus sequence typing (MLST) amplifications were attempted on a subset of positive samples. Three major MLST schemes exist for *Leptospira* spp. typing worldwide, all supported by the online database http://pubmlst.org/leptospira/ [25,27,28]. MLST was attempted for *glmU*, *pntA*, *sucA*, *tpiA*, *pfkB*, *mreA*, and *caiB* genes, i.e. following typing scheme #1 [27]. This scheme was chosen to allow the comparison with published strains from the Pacific region, which used the same targets.

Similarly to the detection of *Leptospira* spp., we used a RT-PCR methodology to increase the sensitivity [20,29] for the *Leptospira* genotyping. All PCR amplifications were performed in 25 μl containing 2 μl of nucleic acids template, 5 μl of one-step RT-PCR buffer, 1 μl of dNTP mix, 1.5 μl (10 μM) of each primer, 12.75 μl of RNase-free water, 1 μl of RT-PCR enzyme mix and 0.25 μl of RNasin (Promega), using QIAGEN one-step RT-PCR kit (QIAGEN Inc., USA) and the Mastercycler Gradient instrument (Eppendorf, Germany). Primers and PCR conditions are provided as supplementary information (S2 Table).

Sanger sequencing was carried out at ILM on the ABI PRISM 310 Sequence Detection System (Applied Biosytems, USA) or PCR samples were sent to Genoscreen company (France).

### Phylogenetic analysis

Consensus sequences and alignments were performed with Geneious Pro version 5.4 [30]. For MLST analyses, sequence alignments were constructed separately for all seven considered loci. A seven-loci concatenate was generated using SEQMATRIX v1.7.8 [31]. Unique allele identifiers for all seven loci were assigned and corresponding allelic profiles (or sequence types STs) were defined using the established *Leptospira* MLST website (http://pubmlst.org), focusing on MLST scheme #1. Phylogenetic trees were constructed based on the maximum-likelihood (ML) method with 1,000 bootstraps, using PhyML 3.1 [32]. Trees were visualized in FigTree v1.3.1 (http://tree.bio.ecd.ac.uk/). GenBank accession numbers of the sequences produced in the frame of the present study are provided as supplementary information (S3 Table).

## Results

### Rat identification

Three rat species were identified: *Rattus norvegicus* (79/113, 70%), *Rattus rattus* (28/113, 25%) and *Rattus exulans* (6/113, 5%).

### *Leptospira* detection and prevalence in animal specimens

Kidneys from 26.5% (48/181) of pigs were positive for *Leptospira* (Table 1). Pigs carrying leptospires originated from 11 out of the 17 selected herds, with a prevalence varying from 10 to 89% between herds.

**Table 1:** *Leptospira* detection and identification by host species. The prevalence of renal infection by *Leptospira* in farm pigs and rodents is based on RT-qPCR detection results. Infecting *Leptospira* species were determined by PCR, targeting either *secY*, *rrs* (16S) or *LipL32* gene in positive samples. The number of successful amplification for *secY* is provided. Based on the observed diversity within the *secY* gene, MLST amplifications were attempted on a subset of positive samples; the number of successful complete MLST on seven (or six) loci is given.

Source	*Leptospira* prevalence (%)	Identification success (%)	Infecting *Leptospira*	*secY* amplification	7-loci (or 6^a^) MLST
Pig	48/181 (26.5)	30/48 (62.5)	*Li* (n = 30), L* (n = 10)	30	4/14 (5)
*RR*	3/28 (10.7)	2/3 (66.7)	*Li* (n = 2)	2	0/2 (1)
*RN*	19/79 (24.1)	13/19 (68.4)	*Li* (n = 8), *Lb* (n = 5)	12	3/12 (6)
*RE*	1/6 (16.7)	1/1 (100)	*Lb* (n = 1)	1	1/1 (1)
All rats	23/113 (20.4)	16/23 (69.6)	*Li* (n = 10), *Lb* (n = 6)	15	4/15 (8)
Dog	-	2/4 (50)	*Li* (n = 2)	2	0
Human	-	39/51 (76.5)	*Li* (n = 37), *Lb* (n = 2)	33	4/9 (4)

*RR*: *Rattus rattus*;

*RN*: *Rattus norvegicus*;

*RE*: *Rattus exulans*.

*Li*: *Leptospira interrogans*;

*Lb*: *Leptospira borgpetersenii*;

L*: undetermined species closely related to *Leptospira mayottensis* and *Leptospira alexanderi*.

^a^ Amplification and sequencing success for the 6-loci MLST scheme excluding the locus *caiB*.

Kidneys from 20.4% (23/113) of trapped rats were positive for *Leptospira* (Table 1). Positive rats were found at five sites, where three to 34 animals were trapped per location. Prevalence varied between these sites from 8.7 to 67%. No *Leptospira*-positive rats were found in the remaining seven sites (where one to seven rats were trapped per site). The *Leptospira* prevalence varied between rat species: 24.1% in *R*. *norvegicus*, 10.7% in *R*. *rattus*, and 16.7% in *R*. *exulans* (Table 1) but not significantly (Fisher’s exact test, P = 0.31). Interestingly, rat species occurred sympatrically in five sampling sites, including one piggery where specimens from the three *Rattus* species were trapped, and where *Leptospira* detection revealed that all three species were carrying *Leptospira* (one *R*. *rattus*, one *R*. *exulans* and six *R*. *norvegicus*; see S3 Table).

The five sampling sites where both pig and rat samples were collected showed various prevalence results: three sites showed positive prevalences for both species, one site showed zero prevalence for both species, and the last piggery revealed one infected pig (out of ten) but no infected rat (out of seven). Detailed *Leptospira* prevalences per site and host species are provided as supplementary information (S3 Table).

Prevalence could not be assessed for dogs or humans, as only positive samples were included in the study.

### *Leptospira* species identification

From the 51 positive human samples, *Leptospira* species were successfully identified in 39 samples (76.5%): *Leptospira interrogans* in 37 cases and *Leptospira borgpetersenii* in two cases (Table 1).

**Fig 3:**
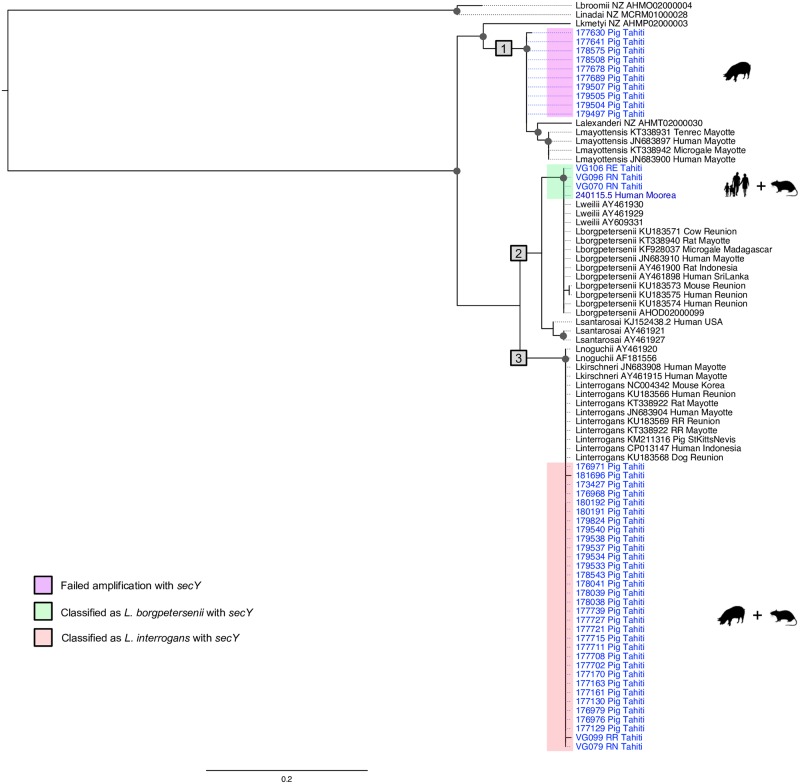
Maximum-likelihood phylogenetic tree (model TN93+I; 1,000 replicates) inferred from *LipL32* gene (229-bp sequence). Clinical and animal samples from Tahiti are shown in light blue, clinical samples from other islands in French Polynesia (FP) in dark blue; referred to using identifiers accompanied by the host name. Corresponding GenBank accession numbers are reported in S5 Table. Published sequences included in the phylogeny are shown in black using the identified *Leptospira* species followed by GenBank accession numbers, the host species and the country of origin. The major genetic groups are highlighted with grey boxes (numbered 1 to 3). Bootstrap values higher than 70% are indicated by a dark circle. Black silhouettes represent host groups from FP (i.e. human, dog, pig or rat).

From the 48 positive pig kidneys, 30 samples (62.5%) were successfully identified as infected by *L*. *interrogans* (Table 1). For the remaining samples, PCR amplification of *secY*, *rrs* (16S) or MLST genes was not successful, but a 241-bp fragment of the *LipL32* gene was sequenced from ten additional samples. The obtained *LipL32* sequences (40 in total) were included in a ML tree (Fig 3) and showed a well-marked separate cluster closely related to *Leptospira mayottensis* and *Leptospira alexanderi*. PCR failure or a lack of nucleic acids template prevented *Leptospira* identification from the eight remaining swine positive samples.

From the 23 positive rat kidneys, *Leptospira* species were successfully identified in 16 samples: *L*. *interrogans* in 10 cases and *L*. *borgpetersenii* in six cases. *Rattus rattus* was infected by *L*. *interrogans*, *Rattus exulans* by *L*. *borgpetersenii* and *Rattus norvegicus* infected with either of the two *Leptospira* species (Table 1).

From the dog specimens, *L*. *interrogans* was successfully identified from two animals.

### Genetic diversity based on *secY* gene and MLST

**Fig 4:**
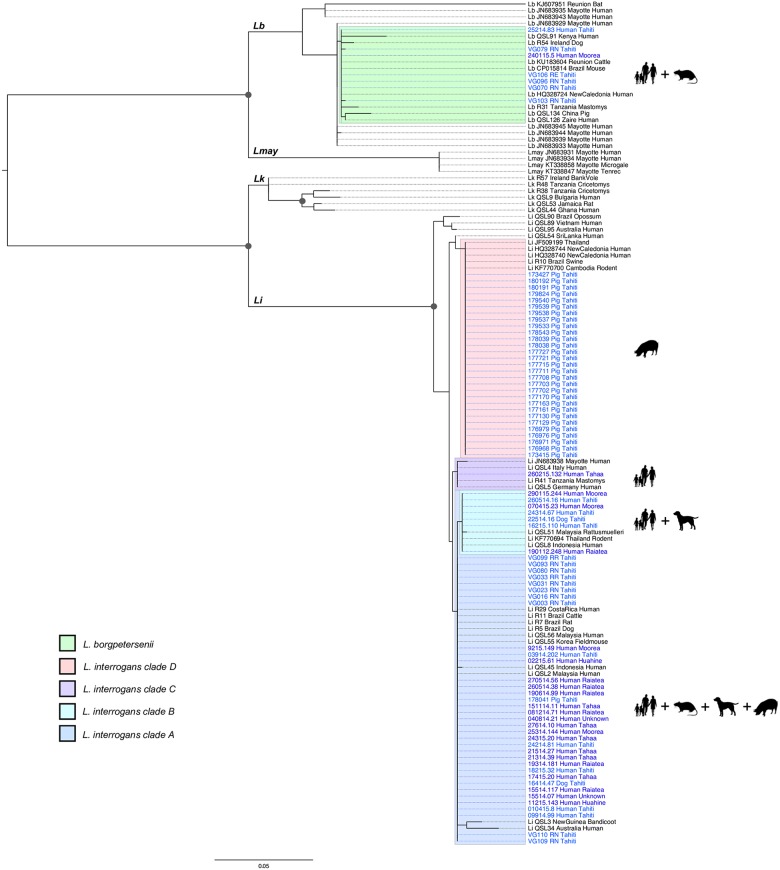
Maximum-likelihood phylogenetic tree (model TN93+G; 1,000 replicates) inferred from *secY* gene (444-bp sequence). Human and animal samples from Tahiti are shown in light blue and clinical samples from other islands in French Polynesia (FP) in dark blue; they are referred to using identifiers accompanied by the host name. Corresponding GenBank accession numbers are reported on S6 Table. Published sequences included in the phylogeny are written in black using GenBank accession numbers or ID (for samples from [33]) followed by the country of origin and the host species. *Leptospira* species are indicated for published sequences: *Li*: *Leptospira interrogans; Lb*: *Leptospira borgpetersenii; Lk*: *Leptospira kirschneri; Lmay*: *Leptospira mayottensis*. Bootstrap values higher than 70% are indicated by a dark circle. Black silhouettes represent host groups from FP (i.e. human, dog, pig or rat). Legend refers to the different clusters including samples from FP, with four clades (A to D) inside the species *Leptospira interrogans*.

Diversity analyses were conducted on a 444-bp length fragment of the *secY* gene including 30 sequences from pigs, 15 from rats, two from dogs and 32 from humans (one human sample was removed from the 33 available because of its shorter sequence; Table 1). The maximum likelihood phylogenetic tree showed four *L*. *interrogans* clusters, as shown in Fig 4. Most of the clinical cases (n = 23), 10 rats, one pig and one dog clustered together into the main clade A. Human cases were from various geographical origins from Society Islands: Tahiti (n = 5), Moorea (n = 2), Raiatea (n = 6), Huahine (n = 2) and Tahaa (n = 6); two were from unknown origin in FP. A second cluster B included clinical samples from Tahiti (n = 3), Moorea (n = 2) and Raiatea (n = 1) and one dog sample. The third cluster C included one clinical sample from Tahaa. Finally, the last *L*. *interrogans* cluster D included pigs only. Almost all pigs (n = 29) clustered together in clade D, except for one pig sample that belonged to the clade A. *Leptospira borgpetersenii* was identified from the remaining five rat and two human samples (one from Tahiti and one from Moorea), forming a single cluster showing the same 444-bp *secY* sequence except for two sequences from rats which differed by one nucleotide each (Fig 4).

For four human samples, eight rats and five pigs, six to seven loci of the MLST scheme were successfully amplified, sequenced and concatenated for subsequent analyses (Table 2). The samples that allowed successful amplification at all MLST loci and identified as *L*. *interrogans*, were distributed in two clusters corresponding to previously described sequence types: ST17 was found in human and rats (corresponding to clade A in the *secY*-based phylogeny) and ST140 in pigs (corresponding to clade D). The samples that allowed successful amplification at all MLST loci and identified as *L*. *borgpetersenii*, corresponded to ST149 (one human and one rat).

**Table 2:** MLST results by host species. For each of the 17 samples referred to by a unique identifier (ID), the table provides the exact or most probable sequence type (ST or allelic profile), the infecting *Leptospira* species and the presumptive serovar associated with the ST. GenBank accession numbers of sequences for each of the seven MLST loci are reported in the supplementary information (S4 Table).

STs	*Leptospira* spp.	Presumptive serovars^a^ (host, country)	ID	Host species
17	*L*. *interrogans*	Icterohaemorrhagiae (human, Japan Belgium) Copenhageni (human, Brazil; rat, Denmark Russia)	40814.21	*Homo sapiens*
			17415.20	*Homo sapiens*
			21514.27	*Homo sapiens*
			VG023*	*R*. *norvegicus*
			VG031*	*R*. *norvegicus*
			VG033*	*R*. *rattus*
			VG080	*R*. *norvegicus*
			VG093	*R*. *norvegicus*
			VG109	*R*. *norvegicus*
140	*L*. *interrogans*	Pomona (human, Australia) Grippotyphosa (human, Sri Lanka) Guaratuba (possum, Brazil)	177702*	*Sus scrofa*
			179533	*Sus scrofa*
			179540	*Sus scrofa*
			179824	*Sus scrofa*
			180192	*Sus scrofa*
149	*L*. *borgpetersenii*	Ballum (mouse, Denmark) Castellonis (mouse, Spain)	240115.5	*Homo sapiens*
			VG070*	*R*. *norvegicus*
			VG106	*R*. *exulans*

STs: sequence types.

^a^ The presumptive serovars corresponding to STs are based on information from the PubMLST database (http://pubmlst.org/leptospira/).

*Samples with incomplete MLST for which the nearest ST match is drawn from available loci and other complete MLST.

## Discussion

Our study provides the first description of circulating *Leptospira* species and genotypes in humans and potential animal reservoirs and/or carriers of leptospires pathogenic for humans in FP. This region presents a low diversity of pathogenic *Leptospira* compared to continental countries, but a similar diversity as found in other tropical islands like New Caledonia (Pacific) [34] or Reunion Island (Indian Ocean) [29] where the same species, i.e. *L*. *interrogans* and *L*. *borgpetersenii*, were identified in human and animal samples. *Leptospira interrogans* was the dominant species, found in 95% of human cases for which the infecting species could be genotypically determined. This is consistent with previous serological surveys showing the predominance of Icterohaemorrhagiae in patients diagnosed in FP [8].

Rodents, especially rats, are recognized as one of the most significant reservoir of leptospires worldwide [2,35]. Our results give the first direct confirmation that rats are actual reservoirs of two pathogenic *Leptospira* species (*L*. *interrogans* and *L*. *borgpetersenii*), the two same species identified from human leptospirosis cases in FP. The *Leptospira* prevalence in rats reported in our study (20.4%) is similar to rates reported in New Caledonia (20.1%) [36] or Mayotte (Indian Ocean) (15.9%) [37], but contrasting with other tropical islands like Reunion island (36.3%) [29] or Seychelles (Indian Ocean) (7.7%) (Léon Biscornet, pers. comm.). Our study also gives the first description of rat species present in Tahiti, confirming the occurrence of all three species (*R*. *rattus*, *R*. *norvegicus* and *R*. *exulans*) that were suspected to be present based on rodent surveys from other FP Islands, i.e. Huahine, Raiatea, Marquesas [16] and Tetiaroa atoll [38], where one to three of the same three *Rattus* spp. were previously identified. *Leptospira* prevalences obtained in our study varied between rat species and sampling sites, but zero prevalences corresponded to sites where the number of trapped rats was low (two to seven), possibly leading to a bias. In particular, rats were difficult to trap in urban areas (high incidence of bait taken with no captures), and *R*. *exulans* specimens (n = 6) were captured from only two sites. Results from the site where all three rat species were present and found infected do not seem to indicate a clustering associated with rat species: *L*. *interrogans* was genotyped from one *R*. *rattus* and four *R*. *norvegicus*, and *L*. *borgpetersenii* was genotyped from one *R*. *exulans* and two *R*. *norvegicus*.

The results of our investigations in slaughtered pigs originating from 16 farms on the islands of Tahiti and Moorea suggest that *Leptospira* infection is quite common among fattened pigs (26.5%), with infection prevalence up to 89% in one farm. Our result is similar to *Leptospira* prevalence reported in farmed pigs from rural Ecuador (21.1%) [39] but significantly higher than prevalences reported in New Caledonia (10.2%) [34] or Thailand (7.9%) [40]. A single infected pig was shown to carry the same genotype as found in most human samples (clade A). Of note, this pig originated from a farm that was not part of the rat sampling sites. Most pigs (n = 29) were infected by another genotype (ST140, clade D), which was not found in any human cases or other tested animal species despite the close contact of rats and pigs inside the five farms where both species were sampled. The fact that “working in a piggery” has been previously reported as a risk factor in FP [8] might be related to the density of rats found around the piggeries, rather than to the pigs themselves, as shown in American Samoa [41]. However, ST140 is not restricted to pigs in other countries as it was associated with symptomatic human leptospirosis in New Caledonia [24], Australia and Sri Lanka (http://pubmlst.org/leptospira/) (presumptive serovars Pomona and Grippotyphosa, see Table 2). The lack of matching genotypes between humans/rats and pigs (except for one pig) has to be considered with caution as the pig sampling dates back to 2011–2012, while the human cases included in our study were diagnosed in 2014–2015 (and the rats sampled in 2015). In order to assess the possible impact of this time gap, we investigated further sera from human cases collected in late 2011 and early 2012 (n = 7). Unfortunately, we successfully amplified and genotyped one sample only, isolated in January 2012 from Raiatea (Society Islands); the corresponding *secY* gene sequence was 100% identical to those isolated from human samples collected in 2014–2015 clustering in clade B, thus including no pig samples. The fact that clade D genotype was not identified from humans in FP could also be explained by a recent introduction of this particular genotype concomitantly with an infected pig. The last importation of breeding pigs into Tahiti occurred in 2006, with 156 animals imported from New Zealand (Hervé Bichet, pers. comm.), where pigs are known maintenance hosts for *L*. *interrogans* serovars Pomona and Tarassovi [42,43], but no MLST analysis has been performed in this country. In conclusion, there is currently insufficient evidence to definitively exclude farm pigs as a source of occupational leptospirosis in FP and further studies are required to clarify their role, especially as the genotypes found in domestic pigs in Tahiti have been responsible for human infections in the Asia-Pacific region (see [21] and Table 2).

Using *Lipl32* gene sequencing, we identified a cluster closely related to *Leptospira mayottensis* and *Leptospira alexanderi* including ten pig samples. *Lipl32* is a highly conserved gene [44] with more than 94% amino acid sequence identity across the main pathogenic species *L*. *interrogans*, *L*. *borgpetersenii*, *L*. *kirschneri*, *L*. *noguchii*, *L*. *santarosai*, and *L*. *weilii* [45], and *Lipl32*-based phylogenies generally fail to properly discriminate between these species [25,44,45]. Our *Lipl32*-based phylogeny shows the same clustering of the six species cited above as previously described, with one clutser including *L*. *interrogans*, *L*. *kirschneri*, and *L*. *noguchii* (clade 3, Fig 3), and another cluster including *L*. *borgpetersenii*, *L*. *weilii* and *L*. *santarosai* (clade 2, Fig 3). However, our pig samples are clearly separate from these two main clusters, and are closely related to *L*. *alexanderi* and *L*. *mayottensis* (clade 1, Fig 3). *Leptospira mayottensis*, first described in 2012 from human clinical cases on the tropical island of Mayotte, Indian Ocean [46,47], has been further identified in small mammals in Mayotte [37] and Madagascar [48]. This finding not only shows the potential circulation of an undescribed *Leptospira* species in Tahiti, but also highlights the rich biodiversity of this phylum yet to be discovered.

Results about dogs are weakened by the small sample size. The sampling was fortuitous, with four samples sent from veterinary clinics for biological confirmation of a clinical diagnosis of leptospirosis. Nevertheless, our phylogeny results show that the two dogs for which we identified the infecting *Leptospira* share two *L*. *interrogans* genotypes with human cases from Tahiti (clades A and B; Fig 4). Interestingly, clade B includes six human cases (all originating from Tahiti or Moorea) and one dog, but no rats. Although a more robust sampling design will be required to estimate the prevalence and the genotypic diversity of leptospires in dogs, the finding of *Leptospira* genotypes common to both dogs and humans raises questions about the potential role of dogs as a source for human leptospirosis infections in FP. This hypothesis is supported by previous identifications of serogroup Canicola in human cases from FP [8,49], suggesting that dogs, as the reservoir for this serogroup, have probably been the source of infection. The dogs included in our study were pets, but the population of stray dogs in Tahiti might also be important. If future investigations found that stray dogs were infected with pathogenic leptospires, it would imply that they could significantly contaminate the environment and thus be an indirect source of human infection.

Risk factors for human leptospirosis infections in FP are the same than those reported elsewhere: contact with infected animals, as well as recreational and occupational activities [9,50]. Our study highlights the need to reinforce control measures in the country to prevent human leptospirosis. We showed rodents as the probable main source of human infection, highlighting the control of rat populations as a priority. We also raised questions about the contribution of stray dogs and farm pigs to the local epidemiology of the disease. Future studies should be designed to precisely determine the extent of this contribution, so that control measures can be tailored accordingly. Finally, this study confirms that serotyping alone is insufficient to decipher the transmission patterns and reservoirs species responsible for human infections, and that direct genotyping is an important additional tool for epidemiological studies of leptospirosis.

## Supporting information

S1 Table Comparison of *Leptospira* qPCR results tested with different protocols and two sets of primers (Smythe et al. 2002 [21] versus Stoddard et al. 2009 [22]). Sensitivity assays included nine rat samples, three human clinical samples, three pig samples and five controls (reference strains).
(XLS)

S2 Table List of primers and conditions used for PCR amplifications of (i) genes used for Leptospira identification at the species level, and (ii) the seven Leptospira loci, with primers previously published by Boonsilp et al. A single multilocus sequence typing (MLST) scheme for seven pathogenic Leptospira species. *PLoS Negl Trop Dis*. 2013;7: e1954.
(DOC)

S3 Table Prevalence of *Leptospira* carriage in animals per site and host species. (*RN*: *Rattus norvegicus; RR*: *Rattus rattus; RE*: *Rattus exulans*).
(DOC)

S4 Table GenBank accession numbers of the nucleotide sequences used in the 7-loci MLST analysis. The 50 sequences from *LipL32* gene and the 79 sequences from *secY* gene are listed in supplementary S5 and S6 Tables respectively.
(DOC)

S5 Table GenBank accession numbers of the nucleotide sequences used in the *Lipl32*-based plylogeny: 50 sequences isolated from pigs (n = 44), humans (n = 1), or rats (n = 5). The sequence lengths range from 198 to 244 bp. Overlapping aligned sequences shorter than 229 bp were removed from the phylogenetic analysis (i.e. pig samples 173415, 177703, 180190 and 181453).
(XLS)

S6 Table GenBank accession numbers of the nucleotide sequences used in the *secY*-based plylogeny (79 sequences isolated from pigs (n = 29), humans (n = 33), dogs (n = 2) or rats (n = 15). The sequence lengths range from 444 to 551 bp. Overlapping aligned sequences shorter than 444 bp were removed from the phylogenetic analysis (i.e. human sample 15414.76).
(XLS)
