# Supplementary material for: Leptospira diversity in animals and humans in Tahiti, French Polynesia
Source: PLoS Negl Trop Dis. 2017 Jun 28;11(6):e0005676. doi: 10.1371/journal.pntd.0005676 (PMC5507467; doi:10.1371/journal.pntd.0005676)
Supplement: S2 Table — A single multilocus sequence typing (MLST) scheme for seven pathogenic Leptospira species. PLoS Negl Trop Dis. 2013;7: e1954. (DOC) [file pntd.0005676.s002.doc]

**Supporting information**

**S2 Table.** **List of primers and conditions used for PCR amplifications** of (i) genes used for *Leptospira* identification at the species level, and (ii) the seven *Leptospira* loci, with primers previously published by Boonsilp et al. A single multilocus sequence typing (MLST) scheme for seven pathogenic Leptospira species. *PLoS Negl Trop Dis*. 2013;7: e1954 (MLST scheme#1).

| **Gene** | **Primers** | **Sequence (5’– 3’)** | **PCR product size (bp)** | **T° annealing** |
| --- | --- | --- | --- | --- |
| *rrs* | LA  LB | GGCGGCGCGTCTITAAACATG  TTCCCCCCATTGAGCAAGATT | 330 | 55°C (down to 51) |
| *secY* | secY-F  secY-R | ATGCCGATCATTTTTGCTTC  CCGTCCCTTAATTTTAGACTTCTTC | 549 | 58°C (down to 53) |
| *lipL32* | LipL32-45F  LipL32-286R | AAGCATTACCGCTTGTGGTG  GAACTCCCATTTCAGCGATT | 241 | 58°C |
| *glmU* | glmU-F  glmU-R | AGGATAAGGTCGCTGTGGTA  AGTTTTTTTCCGGAGTTTCT | 650 | 50°C |
| *pntA* | pntA –F  pntA-R | TAGGAAARATGAAACCRGGAAC  AAGAAGCAAGATCCACAAYTAC | 621 | 52°C or 50°C |
| *sucA* | sucA-F  sucA-R | TCATTCCACTTYTAGATACGAT  TCTTTTTTGAATTTTTGACG | 640 | 52°C |
| *tpiA* | tpiA-F  tpiA-R | TTGCAGGAAACTGGAAAATGAAT  GTTTTACRGAACCHCCGTAGAGAAT | 639 | 52°C or 50°C |
| *pfkB* | pfkB-F  pfkB-R | CGGAGAGTTTTATAARAAGGACAT  AGAACACCCGCCGCAAAACAAT | 588 | 52°C or 50°C |
| *mreA* | mreA-F  mreA-R | GGCTCGCTCTYGACGGAAA  TCCRTAACTCATAAAMGACAAAGG | 719 | 52°C |
| *caiB* | caiB-F  caiB-R | CAACTTGCGGAYATAGGAGGAG  ATTATGTTCCCCGTGAYTCG | 650 | 50°C |

Amplifications were performed using QIAGEN one-step RT-PCR kit (QIAGEN Inc., USA) with 2 µL of DNA in a 23 µl reaction mixture containing 5 µl of RT-PCR reaction mix, 1.5 µl (10 µM) of each primer, 12.75 µl of RNAase free water, 1 µl of reverse transcriptase and 0.25 µl of RNAsine. The PCR conditions consisted of a transcription at 50°C for 30 min and an initial denaturation step at 95°C for 15 min, followed by 45 cycles of denaturation at 94°C for 30 s, annealing between 50-58°C for 30 s, and extension at 72°C for 1 min, with a final extension at 72°C for 10 min.
