# Supplementary material for: Leptospira diversity in animals and humans in Tahiti, French Polynesia
Source: PLoS Negl Trop Dis. 2017 Jun 28;11(6):e0005676. doi: 10.1371/journal.pntd.0005676 (PMC5507467; doi:10.1371/journal.pntd.0005676)
Supplement: S3 Table — (RN: Rattus norvegicus; RR: Rattus rattus; RE: Rattus exulans). (DOC) [file pntd.0005676.s003.doc]

**Supporting information**

**S3 Table.** **Prevalence of *Leptospira* carriage in animals per site and host species.** (*RN: Rattus norvegicus; RR: Rattus rattus; RE: Rattus exulans*)

| Sampling site | *Rattus norvegicus* | *Rattus rattus* | *Rattus exulans* | N rats | Rats prevalence % (positive per species) | N pigs | Pigs prevalence % (n positive) |
| --- | --- | --- | --- | --- | --- | --- | --- |
| Vallée Hamuta | 1 | 2 | 0 | 3 | 66.7 (2 *RR*) | - | - |
| Pointe des pêcheurs | 0 | 2 | 0 | 2 | 0 (0) | - | - |
| ILM Papeete | 0 | 2 | 0 | 2 | 0 (0) | - | - |
| Taravao | 1 | 1 | 0 | 2 | 0 (0) | - | - |
| ILM Paea | 7 | 7 | 0 | 14 | 21.4 (3 *RN*) | - | - |
| Pirae | 0 | 1 | 1 | 2 | 0 (0) | - | - |
| Port Papeete | 2 | 0 | 0 | 2 | 0 (0) | - | - |
| Piggery Tahiti 1 | 1 | 0 | 0 | 1 | 0 (0) | 10 | 0 (0) |
| Piggery Tahiti 2 | 0 | 7 | 0 | 7 | 0 (0) | 10 | 10.0 (1) |
| Piggery Tahiti 3 | 23 | 0 | 0 | 23 | 8.7 (2 *RN)* | 9 | 88.9 (8) |
| Piggery Tahiti 4 | 10 | 6 | 5 | 21 | 38.1 (1 *RR,* 6 *RN,* 1 *RE*) | 15 | 60.0 (9) |
| Piggery Tahiti 5 | 34 | 0 | 0 | 34 | 23.5 (8 *RN*) | 20 | 55.0 (11) |
| Piggery Tahiti 6 | - | - | - | - | - | 9 | 11.1 (1) |
| Piggery Tahiti 7 | - | - | - | - | - | 10 | 30.0 (3) |
| Piggery Tahiti 8 | - | - | - | - | - | 16 | 18.8 (3) |
| Piggery Tahiti 9 | - | - | - | - | - | 10 | 0 (0) |
| Piggery Tahiti 10 | - | - | - | - | - | 3 | 0 (0) |
| Piggery Tahiti 11 | - | - | - | - | - | 10 | 60.0 (6) |
| Piggery Tahiti 12 | - | - | - | - | - | 11 | 0 (0) |
| Piggery Tahiti 13 | - | - | - | - | - | 8 | 12.5 (1) |
| Piggery Tahiti 14 | - | - | - | - | - | 10 | 30.0 (3) |
| Piggery Tahiti 15 | - | - | - | - | - | 10 | 20.0 (2) |
| Piggery Tahiti 16 | - | - | - | - | - | 10 | 0 (0) |
| Piggery Moorea | - | - | - | - | - | 10 | 0 (0) |
| Global | 79 | 28 | 6 | 113 | 20.4 (3 *RR*, 19 *RN,* 1 *RE*) | 181 | 26.5 (48) |
