# Supplementary material for: Leptospira diversity in animals and humans in Tahiti, French Polynesia
Source: PLoS Negl Trop Dis. 2017 Jun 28;11(6):e0005676. doi: 10.1371/journal.pntd.0005676 (PMC5507467; doi:10.1371/journal.pntd.0005676)
Supplement: S4 Table — The 50 sequences from LipL32 gene and the 79 sequences from secY gene are listed in supplementary S5 and S6 Tables respectively. (DOC) [file pntd.0005676.s004.doc]

**Supporting information**

**S4 Table.** **GenBank accession numbers of the nucleotide sequences used in the MLST analysis.** The 50 sequences from *LipL32* gene and the 79 sequences from *secY* gene are listed in supplementary tables S5 and S6 respectively.

| **ID** | **Host species** | **Tissue type** | **FP Island** | **MLST loci** | | | | | | | ***secY*** |
| --- | --- | --- | --- | --- | --- | --- | --- | --- | --- | --- | --- |
|  |  |  |  | *glmU* | *pntA* | *sucA* | *tpiA* | *pfkB* | *mreA* | *caiB* |  |
| 177702 | *Sus scrofa* | Kidney | Tahiti | KY356901 | KY357002 | KY357019 | KY357036 | KY356985 | KY356968 | - | KY357092 |
| 179533 | *Sus scrofa* | Kidney | Tahiti | KY356902 | KY357003 | KY357020 | KY357037 | KY356986 | KY356969 | KY356889 | KY357102 |
| 179540 | *Sus scrofa* | Kidney | Tahiti | KY356903 | KY357004 | KY357021 | KY357038 | KY356987 | KY356970 | KY356890 | KY357106 |
| 179824 | *Sus scrofa* | Kidney | Tahiti | KY356904 | KY357005 | KY357022 | KY357039 | KY356988 | KY356971 | KY356891 | KY357107 |
| 180192 | *Sus scrofa* | Kidney | Tahiti | KY356905 | KY357006 | KY357023 | KY357040 | KY356989 | KY356972 | KY356892 | KY357109 |
| 240115.5 | *Homo sapiens* | Serum | Moorea | KY356906 | KY357007 | KY357024 | KY357041 | KY356990 | KY356973 | KY356893 | KY357112 |
| 40814.21 | *Homo sapiens* | Serum | - | KY356907 | KY357008 | KY357025 | KY357042 | KY356991 | KY356974 | KY356894 | KY357077 |
| 17415.20 | *Homo sapiens* | Serum | Tahaa | KY356908 | KY357009 | KY357026 | KY357043 | KY356992 | KY356975 | KY356895 | KY357065 |
| 21514.27 | *Homo sapiens* | Serum | Tahaa | KY356909 | KY357010 | KY357027 | KY357044 | KY356993 | KY356976 | KY356896 | KY357069 |
| VG023 | *Rattus norvegicus* | Kidney | Tahiti | KY356910 | KY357011 | KY357028 | KY357045 | KY356994 | KY356977 | - | KY357119 |
| VG031 | *Rattus norvegicus* | Kidney | Tahiti | KY356911 | KY357012 | KY357029 | KY357046 | KY356995 | KY356978 | - | KY357120 |
| VG033 | *Rattus rattus* | Kidney | Tahiti | KY356912 | KY357013 | KY357030 | KY357047 | KY356996 | KY356979 | - | KY357121 |
| VG070 | *Rattus norvegicus* | Kidney | Tahiti | KY356913 | KY357014 | KY357031 | KY357048 | KY356997 | KY356980 | - | KY357122 |
| VG080 | *Rattus norvegicus* | Kidney | Tahiti | KY356914 | KY357015 | KY357032 | KY357049 | KY356998 | KY356981 | KY356897 | KY357124 |
| VG093 | *Rattus norvegicus* | Kidney | Tahiti | KY356915 | KY357016 | KY357033 | KY357050 | KY356999 | KY356982 | KY356898 | KY357125 |
| VG106 | *Rattus exulans* | Kidney | Tahiti | KY356916 | KY357017 | KY357034 | KY357051 | KY357000 | KY356983 | KY356899 | KY357129 |
| VG109 | *Rattus norvegicus* | Kidney | Tahiti | KY356917 | KY357018 | KY357035 | KY357052 | KY357001 | KY356984 | KY356900 | KY357130 |
